# Transarterial chemoembolisation in patients with hepatocellular carcinoma: low-dose doxorubicin reduces post-embolisation syndrome without affecting survival—prospective interventional study

**DOI:** 10.1186/s41747-021-00204-6

**Published:** 2021-03-02

**Authors:** Ahmed A. Bessar, Ahmed Farag, Sameh M. Abdel Monem, Fady M. Wadea, Shady E. Shaker, Mahmoud Ahmed Ebada, Manar A. Bessar

**Affiliations:** 1 Department of Radiodiagnosis, Zagazig University School of Human Medicine, Zagazig, Egypt; 2 Department of Surgery, Zagazig University School of Human Medicine, Zagazig, Egypt; 3 Department of Tropical Medicine, Zagazig University School of Human Medicine, Zagazig, Egypt; 4 Department of Internal Medicine, Zagazig University School of Human Medicine, Zagazig, Egypt; 5 Department of Radiodiagnosis, National Hepatology and Tropical Medicine Research Institute, Cairo, Egypt

**Keywords:** Chemoembolization (therapeutic), Doxorubicin, Carcinoma (hepatocellular), Hepatitis

## Abstract

**Background:**

No chemotherapeutic agents have been standardised for transarterial chemoembolisation (TACE). In particular, doxorubicin has no defined optimal dosage in TACE procedures. We compared low *versus* currently used dose of doxorubicin for TACE in patients with hepatocellular carcinoma (HCC) in terms of severity of post-embolisation syndrome (PES) and overall survival (OS).

**Methods:**

From October 2014 to March 2018, we enrolled patients with primary HCC scheduled for TACE. Patients were randomised to receive 50 mg (group A) or 100 mg (group B) of doxorubicin. Outcomes were the rate of patients with PES; free-time-to-PES; changes in laboratory results; tumour response at 1, 3, and 6 months after TACE; and overall survival.

**Results:**

Twenty-eight patients (24 males, 4 females) were enrolled, aged 58.9 ± 6.8 years (mean ± standard deviation). Fifteen of them palliated with 50 mg (group A) and 13 with 100 mg (group B) of doxorubicin for a total of 68 TACE procedures (of 28 patients who had repeated TACE procedures). Visual analogue scale (VAS) and duration of pain were significantly differently lower in group A than in group B (*p* < 0.001). The median duration of fever was shorter in group A than in group B (*p* = 0.003). No significant differences between both groups were observed for tumour response to TACE and OS. The doxorubicin dose was significantly correlated with duration of pain, fever, and VAS score.

**Conclusion:**

A lower dose of doxorubicin (50 mg) was associated with fewer PES symptoms compared with 100 mg, without effects on tumour response nor OS.

## Key points

- In transarterial chemoembolisation of hepatocellular carcinoma (HCC), 50 mg doxorubicin was associated with fewer post-embolisation syndrome symptoms than 100 mg doxorubicin.
- The doxorubicin dose was positively correlated with post-injection visual analogue scale (VAS) score, fever days, and duration of pain after the procedure.

## Background

Hepatocellular carcinoma (HCC) is the most common visceral malignancy, it represents around 90% of primary liver cancers worldwide, and it is the second leading cause of cancer-related deaths globally [1–4].

The only radical curative management for HCC is liver transplant; however, this option of treatment is not feasible for most cases, and a partial hepatic resection is an acceptable option. Recently, several interventional alternatives such as the local thermal ablation including radiofrequency and microwave ablation, and transarterial embolotherapy such as transarterial hepatic chemoembolisation (TACE) have been introduced for unresectable HCC [4, 5].

Most HCC cases are inoperable because of the size, location, and number of lesions, as well as poor liver function in most patients. Therefore, TACE has been widely used as a treatment for unresectable HCC and recommended for patients with an intermediate stage (B) [1, 2, 6–9]. Cellular destruction in cases of TACE and systemic release of anti-cancer drug are usually complicated by post-embolisation syndrome (PES) that is manifested by constitutional symptoms followed by patients’ discomfort. The most common complication of TACE is PES consisting of nausea, vomiting, pain in the right upper quadrant, and fever in most patients. PES is typically accompanied by an elevation of hepatic enzymes. PES is responsible for increasing both the patients’ TACE-related length of stay and recurrent readmissions [10–12].

Despite the advances and technical progress in the past few years in diagnosis and management for HCC, the long-term survival of patients managed with TACE is not satisfactory, mainly due to the high rates of tumour recurrence. A cumulative meta-analysis of the studies has clearly shown that the 2-year survival of patients with unresectable HCC who underwent arterial embolisation or chemoembolisation has improved compared with conservative management [1, 13]. In TACE procedures, since the PRECISION V study, doxorubicin has been extensively used as a chemotherapeutic agent in TACE procedures [14].

In the literature, there is no optimal dosage of doxorubicin in TACE procedures (ranging from 30 to 75 mg/m^2^ up to a maximum of 150 mg/m^2^) [15, 16]. According to the recent Asia-Pacific Clinical Practice Guidelines on the management of HCC, chemoembolisation using drug-eluting beads has similar therapeutic efficacy with less systemic adverse events compared with conventional TACE [4].

In this study, we aimed to compare the effects of low *versus* currently used doses of doxorubicin in TACE on PES and the overall survival (OS) in patients with advanced HCC.

## Methods

After obtaining Institutional Review Board’s approval (IRB#:6551-18-11-2018) and in accordance with the ethical principles (as revised in 2013) of the Helsinki Declaration, this study was conducted [17]. This study is a single-institution, prospective interventional study during the period between October 2014 and March 2018 on patients diagnosed with primary HCC on top of cirrhotic hepatitis who underwent chemoembolisation as a treatment. Twenty-nine patients were randomised into two groups: (i) group A where only 50 mg of doxorubicin was injected with the prepared emulsion and (ii) group B where the injected emulsion contained 100 mg of doxorubicin. In group B, one patient was lost to follow-up within the first month so not included in the results.

### HCC diagnosis of and treatment

For the diagnosis of HCC, conclusions of the Barcelona-2000 EASL Conference for Surveillance and recall strategy for HCC were used [18]. The treatment alternatives, procedure details, results, and complications in all cases undergoing TACE during a multidisciplinary tumour board meeting were fully discussed, and a written consent is obtained from the patients. For each patient, complete multiphase computed tomography (CT) assessment including assessment of number, size, and appearance of the tumour(s) was performed. The summation of the diameters in case of more than one lesion is calculated for each patient.

### Eligibility criteria

In this study, the patients with intermediate-stage HCC according to the BCLC staging classification (*i.e*., BCLC stage B) who underwent successful TACE procedures were included. All patients in this study had HCC on top of hepatitis C virus-related cirrhosis, aged 40–80 years, and with serum creatinine level < 1.5 mg/dL (< 132.6 μmol/L) and International normalised ratio (INR) ≤ 1.5. Patients with resectable masses, who were not candidates for local thermal ablation but refusing surgery, were included in this study.

The exclusion criteria included (1) early-stage HCC according to BCLC [1], (2) concurrent serious medical condition(s) such as underlying cardiac or end-stage renal disease, (3) history of concurrent primary malignancies, (4) recent history of encephalopathy, (5) presence of uncontrollable ascites, (6) thrombosis in the main stem of the portal vein or one of its major branches, (7) total bilirubin level > 2 mg/dL (> 34.2 μmol/L), and (8) presence of obvious arterioportal shunts observed in triphasic CT or angiographic study.

### Sample size calculation

The sample size was calculated on the basis of the results of Kalva et al. [8] of overall survival in HCC patients who underwent TACE procedures with doxorubicin. By the results of this study, the study population consisted of 29 patients which formed the final sample size to be recorded for the study according to the following equation:

$$ \mathrm{Sample}\ \mathrm{size}=\mathrm{Z}1-a/{2}^2p\left(1-p\right)/{d}^2.\mathrm{Z}1-a/2=1.96,p=\left(32\%\right),\mathrm{and}\ d=0.05 $$

### Randomisation

All patients were randomised one-to-one to undergo Lipiodol chemoembolisation with either 50 or 100 mg doxorubicin in the injected mixture. We utilised an open-source Internet randomisation software (www.randomisation.com) to make a 1:1 allocation list. Alphanumeric identification codes were assigned to the patients to be randomised to one of the two treatments; then, each was kept in a separate envelope.

### Procedure

The preprocedure assessment included the medical history, clinical evaluation, and laboratory findings with the assessment of Child-Pugh grading. Full laboratory data included complete blood picture, liver enzymes, total and direct bilirubin levels, total protein level, albumin level, uric acid level, creatinine level, INR, prothrombin time, and partial thromboplastin time. At 12–24 h preprocedure, 8 mg of dexamethasone was intramuscularly injected [11, 19]. All TACE procedures were performed according to the standard protocol [7, 9].

Procedures were scheduled with not less than a 3-month gap between two sessions, to a maximum of three sessions per patient. All procedures were performed under local anaesthesia and conscious sedation. A femoral puncture was performed using the Seldinger technique. A 4- or 5-French Cobra or SIM II catheter was advanced under fluoroscopy. Prior to embolisation, angiography of the hepatic and mesenteric arteries was performed to map the liver vascular anatomy, to check for arterioportal shunts, and to identify the pathological arterial feeders of the tumour(s). Angiography was performed with a non-ionic contrast agent iohexol (Omnipaque, General Electric Healthcare, Princeton, NJ, USA). A mixture of 10 mL of Lipiodol mixed with doxorubicin dissolved in 5 mL of the contrast was prepared [7].

The injected emulsion was prepared by using the three-way stopcock method with glass or polycarbonate syringes. The content of the syringe loaded with the drug/water-soluble solution should be first pushed towards the syringe containing Lipiodol to favour a water-in-oil emulsion by inducing large drops of the drug within Lipiodol; then, vigorous mixing of the chemotherapy aqueous solution and Lipiodol via the three-way stopcock was done to generate a sufficient energy that decreases the size of droplets. At least 20 pumping exchanges are needed to obtain the smallest possible size of droplets [7, 14]. The injection was made slowly to avoid inadvertent extra-hepatic escape of the mixture, and 2.4- or 2.8-French microcatheter was used for more superselective chemoembolisation and to reduce the PES. The primary endpoint for embolisation is the stasis of the blood flow towards the blush [11, 16]. In cases of early emulsion backflow, we waited for a few minutes to allow more distal concentration; then, the remaining dose was injected.

The concentration of Lipiodol within the tumour was evaluated by cone-beam CT that was performed during and after the injection of the emulsion. This provided an immediate assessment of the tumour coverage by Lipiodol which is a radio-opaque contrast agent. In fact, evaluation of Lipiodol deposition is essential as a predictor factor for tumour response and OS [20].

Before the procedure, each patient received 50 mg of pethidine with maintained intravenous normal saline for continuous hydration. Post-procedure medications were prescribed to reduce the possibility of PES. In addition, each patient received 8 mg of ondansetron for chemotherapy-induced nausea [21].

### Post-procedural follow-up

#### Clinical follow-up and outcome assessment

The clinical condition was assessed at 6 and 24 h, then weekly for 1 month post-procedure. The retrieved clinical data included all complications of PES including abdominal pain, fever, vomiting, nausea, and anorexia. The pain was assessed based on a visual analogue scale (VAS), which is a patient-rated outcome that has been validated for pain. The VAS score was a 10-cm line scored by the patient based on how intense he/she thinks the pain is, where ‘0’ implies very well tolerated and ‘10’ implies very poorly tolerable, and it was calculated with the help of VAS [22].

In this study, we used the Common Terminology Criteria for Adverse Events v5.0 [23] as a standard way to report complications. Regarding fever, it was graded as follows: grade 1 (38.0–39.0 °C), grade 2 (> 39.0–40.0 °C), grade 3 (> 40.0 °C for ≤ 24 h), and grade 4 (> 40.0 °C for > 24 h). Vomiting was graded as follows: grade 1 (no intervention needed), grade 2 (required medication and intravenous hydration), grade 3 (required tube feeding and parenteral nutrition), and grade 4 as a life-threatening condition due to severe persistent vomiting.

Laboratory assessment should be at baseline (< 30 days before the procedure), acute period (0–30 days after the procedure), and chronic period (30–90 days after the procedure). The level of hepatic enzymes (aspartate aminotransferase [AST] and alanine aminotransferase [ALT]) and INR were calculated by any increase from basal > 25% and were recorded. Regarding hyperbilirubinemia, any increase of more than 50% was recorded. OS status was reported, and it was defined as the time from TACE procedure to death from any cause. Patients were considered alive at the last date known and censored at that date [19, 24, 25].

#### Radiological evaluation of the treatment efficacy

All patients were asked for a 1-week post-procedure visit to perform pelvi-abdominal ultrasound. Then, at a 1-month follow-up, the radiological response was assessed by contrast-enhanced CT (contrast-enhanced dynamic magnetic resonance imaging was requested in case of equivocal results) [3, 20]. The successful radiological response was achieved if there was a sufficient Lipiodol concentration inside the lesion with the absence of any enhanced lesions in the arterial phase in contrast-enhanced CT. Radiological evaluation was performed for each patient after TACE by 4 weeks according to the Modified Response Evaluation Criteria in Solid Tumors (mRECIST) criteria [26, 27] and responses being assessed into complete response (CR), partial response (PR), stable disease (SD), or progressive disease (PD). As TACE was repeated every 3 months, the endpoint for TACE was either achievement of CR, deteriorated clinical condition, or extrahepatic disease. The maximum number of TACE procedures were three/patient [26, 27]. The next CT follow-up exams were either after any TACE procedure by 4 weeks or every 3 months after CR. In cases of recurrent activity of the chemoembolised tumour(s) after CR, another session of TACE was performed.

### Data analysis

Data were fed to the computer and analysed using the IBM SPSS software package version 20.0. (Armonk, NY: IBM Corp.). The Kolmogorov–Smirnov was used to verify the normality of distribution of variables. Comparisons between the groups for categorical variables were assessed using the chi-square test (Fisher exact or Monte Carlo correction). The Student *t* test was used to compare two groups for normally distributed quantitative variables while the Mann–Whitney test was used to compare between two groups for not normally distributed quantitative variables. The Friedman test was used to compare between more than two periods for more than two categories. The Kaplan–Meier survival curve was used for the significant relation with progressive-free disease. Significance of the obtained results was judged at the 5% level.

## Results

### Characteristics of the study population

Sixty-eight TACE procedures were performed in 28 HCC patients, the mean age of group A was 60.8 ± 6.4 years and of group B was 56.6 ± 6.8 years, at the time of the first procedure with no statistically significant differences between both groups. Of the included patients, 24 patients were males (85.7%), and 27 (96.4%) patients had repeated TACE (two times in 50% and three times in 46.4% of the patients) (Table 1). The whole emulsion was injected in 60 procedures (60/68, 88%); seven procedures required additional arterial occlusion to the residual feeding vessels using gel foam. More than 90% of the emulsion was injected in the remaining eight procedures. No more Lipiodol was injected in any procedure.

**Table 1:** Demographic and imaging characteristics of patients in groups A and B

	Group A (***n*** = 15)	Group B (***n*** = 13)	Statistical test	***p*** value
**Age (years)**				
Mean ± standard deviation	60.8 ± 6.4	56.6 ± 6.8	*t* = 1.674	0.106
Median (minimum–maximum)	60 (53–74)	55 (44–66)		
**Gender**				
Male	12 (80%)	12 (92.3%)	*χ*^2^ = 0.862	0.600 (FE)
Female	3 (20%)	1 (7.7%)		
**Number of TACE procedures**				
1	1 (6.7%)	0 (0%)	*χ*^2^ = 1.202	0.842 (MC)
2	8 (53.3%)	6 (46.2%)		
3	6 (40%)	7 (53.8%)		
**Number of lesions per patient**				
1	6 (40%)	4 (30.8%)	*χ*^2^ = 0.776	0.871 (MC)
2	8 (53.3%)	7 (53.8%)		
3	1 (6.7%)	2 (15.4%)		
**Pattern of masses in CT/MRI**				
Irregular	8 (53.3%)	5 (38.5%)	*χ*^2^ = 0.619	0.431
Well-defined	7 (46.7%)	8 (61.5%)		
**Size of masses in cm**				
Mean ± standard deviation	7.4 ± 2.2	6.7 ± 2.1	*t* = 0.854	0.401
Median (minimum–maximum)	7 (4–11)	7 (3–11)		

*FE* Fisher exact, *MC* Monte Carlo, *t* Student *t* test, *p p* value for comparing between the studied groups

The most common HCC pattern was “well-defined” lesion (15/28, 53.6%). Group A contained 15 patients who underwent 36 procedures using 50 mg of doxorubicin, while group B consisted of 13 patients who underwent 32 procedures using 100 mg of doxorubicin. The mean size of the masses was 7.4 ± 2.2 cm (mean ± standard deviation) in group A and 6.7 ± 2.1 cm (mean ± standard deviation) in group B, without significant difference (*p* = 0.401). Basic characteristics are shown in Table 1.

### Symptoms and laboratory results

All patients in this study suffered from symptoms of PES (Table 2). Regarding VAS, there was a significant difference between both groups (*p* < 0.001): the median VAS was 4 (interquartile range [IQR] 2) in group A and was 7 (IQR 1) in group B participants. A significant difference was found between the groups regarding the median fever days (*p* = 0.003): the median fever days was 5 days (IQR 2) in group A and 10 days (IQR 5) in group B participants. Regarding vomiting, no significant difference between both groups (*p* = 0.193), and according to the Common Terminology Criteria for Adverse Events v5.0, all patients in this study suffered only from either grade 1 or 2 vomiting. No patient required hospitalisation or specific intervention due to vomiting. The free-time-to-PES manifestations were significantly shorter in group A (7.2 ± 3.6 days, mean ± standard deviation) than in group B (14.5 ± 4.5 days, mean ± standard deviation) (*p* < 0.001). Regarding the occurrence of ascites in the first month, there was no statistically significant difference between both groups (*p* = 0.5). Also, there are no statistically significant differences between both groups about the changes in ALT, AST, total bilirubin, and INR levels at the first and third months.

**Table 2:** Comparison between groups A and B according to clinical outcome and overall survival

	Group A	Group B	***p*** value
**Average VAS score at the first week**			
Mean ± standard deviation	3.9 ± 1	6.6 ± 0.7	< 0.001
Median (minimum–maximum)	4 (3–6)	7 (6–8)	
**Fever days,** ***n*** **(%)**			
**< 5 days**	8 (38.1%)	2 (11.1%)	0.004
**5–10 days**	12 (57.1%)	7 (38.9%)	
** > 10 days**	1 (4.8%)	9 (50%)	
**Fever days, median (interquartile range)**	5 (2)	10 (5)	0.003
**Free-time-to-PES symptoms (days)**			
Mean ± standard deviation	7.2 ± 3.6	14.5 ± 4.5	< 0.001
Median (minimum–maximum)	6 (3–17)	14 (5–21)	
**Ascites occurrence, number of patients (%)**	2 (13.3%)	3 (23.1%)	0.5
**Frequency of ALT levels elevation**			
At month 1	9 (60%)	12 (92.3%)	0.084 (FE)
At month 3	7 (46.7%)	8 (61.5%)	0.431
**Frequency of AST levels elevation**			
At month 1	11 (73.3%)	8 (61.5%)	0.689 (FE)
At month 3	11 (73.3%)	10 (76.9%)	1.000 (FE)
**Frequency of total bilirubin levels elevation**			
At month 1	5 (33.3%)	4 (30.8%)	1.000 (FE)
At month 3	7 (46.7%)	6 (46.2%)	0.978
**Frequency of INR levels elevation**			
At month 1	2 (13.3%)	2 (15.4%)	1.000 (FE)
At month 3	3 (20%)	2 (15.4%)	1.000 (FE)

*ALT* Alanine aminotransferase, *AST* Aspartate aminotransferase, *FE* Fisher exact, *INR* International normalised ratio, *PES* Post-embolisation syndrome, *VAS* Visual analogue scale

### Tumour response and overall survival

While it was statistically not significant, PR was higher in group A than in group B at 1 month (*p* = 0.497). CR was lower in group A than in group B at 1 month (*p* = 0.262) and at 3 months (*p* = 0.280) post-TACE. The mean OS was 14.3 ± 10.6 months (mean ± standard deviation) and 22 ± 12.7 months (mean ± standard deviation) in group A and in group B, respectively, even though statistically not significant (*p* = 0.098) (Table 3).

**Table 3:** Comparison between group A and group B according to mRECIST at follow-up

mRECIST	Group A (***n*** = 15)	Group B (***n*** = 13)	Test of Sig.	***p*** value
**At 1 month**				
CR	2 (13.3%)	4 (30.8%)	*χ*^2^ = 1.512	0.640 (MC)
PR	11 (73.3%)	7 (53.8%)		
SD	2 (13.3%)	2 (15.4%)		
PD	0 (0%)	0 (0%)		
**At 3 months**				
CR	3 (20%)	5 (38.5%)	*χ*^2^ = 4.775	0.250 (MC)
PR	6 (40%)	6 (46.2%)		
SD	0 (0%)	1 (7.7%)		
PD	5 (33.3%)	1 (7.7%)		
Lost at follow-up	1 (6.7%)	0 (0%)		
**At 6 months**				
CR	4 (26.7%)	7 (53.8%)	*χ*^2^ = 3.129	0.325 (MC)
PR	1 (6.7%)	0 (0%)		
SD	1 (6.7%)	0 (0%)		
PD	0 (0%)	0 (0%)		
Lost at follow-up	9 (60.0%)	6 (46.2%)		
**Overall survival**				
< 6 months	3 (20%)	1 (7.7%)	*χ*^2^ = 2.276	0.391 (MC)
6–12 months	7 (46.7%)	4 (30.8%)		
> 12 months	5 (33.3%)	8 (61.5%)		
Mean ± standard deviation	14.3 ± 10.6	22 ± 12.7	*U* = 61.0	0.098
Median (minimum–maximum)	10 (3–33)	23 (3–42)		

*CR* Complete response, *MC* Monte Carlo, *mRECIST* Modified Response Evaluation Criteria in Solid Tumors, *PD* Progressive disease, *PR* Partial response, *SD* Stable disease, *U* Mann–Whitney *U* test

**Fig. 1:**
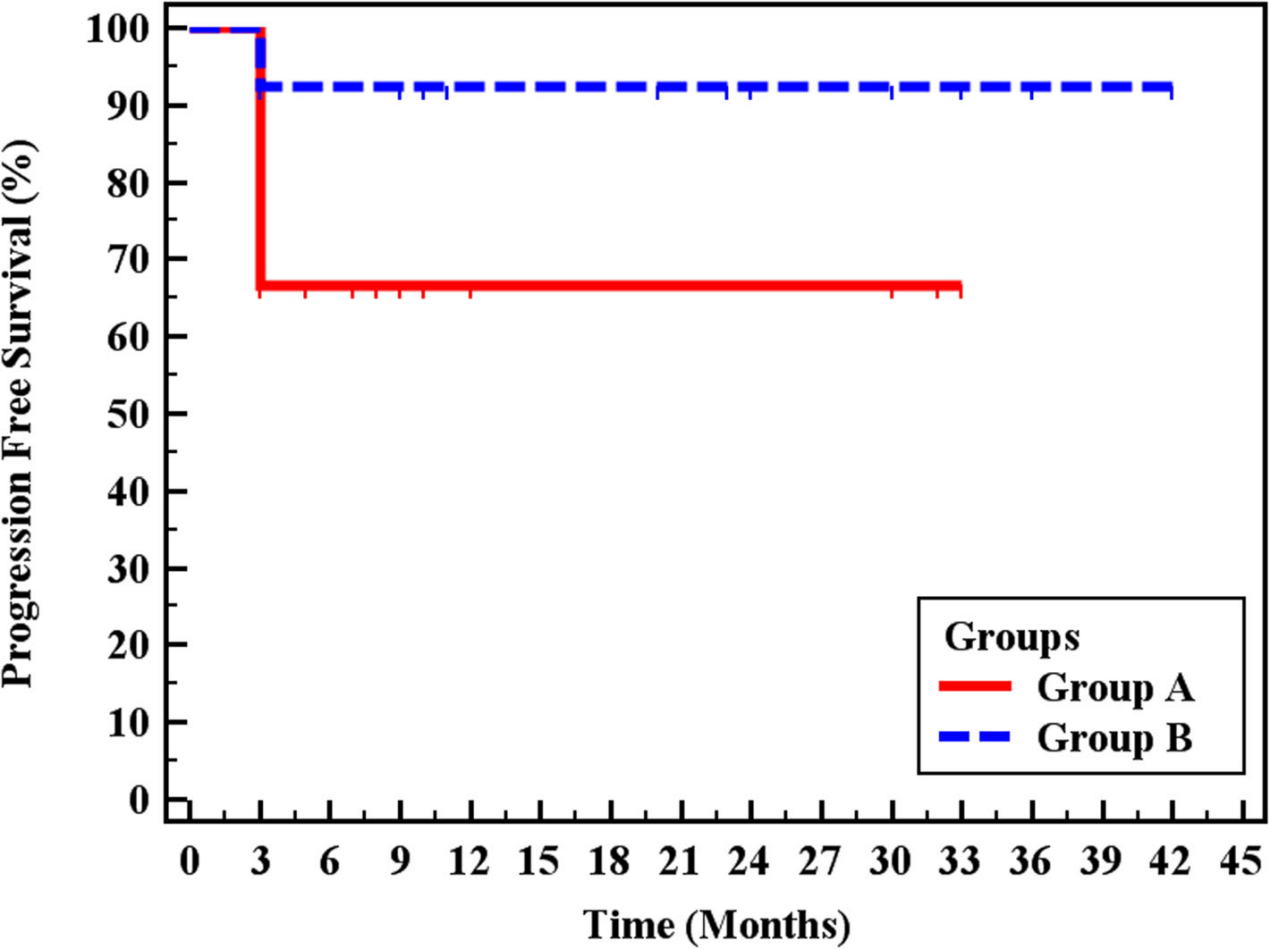
Kaplan–Meier survival curve for progression-free survival for group A and group B

Using the Kaplan–Meier survival curve to analyse whether doxorubicin dose influenced the progression-free survival, we found that the increased doxorubicin dose was not significantly associated with progression-free survival (*p* = 0.105). Progression-free survivals for group A (50 mg) and group B (100 mg) were 66.7 % and 92.3%, respectively (Table 4, Fig. 1)

**Table 4:** Kaplan–Meier survival curves for progression-free survival

	Mean by months	% End of study by months	Log-rank	
			*χ*^2^	*p* value
Groups				
Group A	23.0	66.7	2.622	0.105
Group B	39.0	92.3

## Discussion

TACE is considered the first-choice treatment option for patients with intermediate-stage HCC (BCLB B) with a median survival of 40 months, in well-selected candidates, using a superselective approach [1].

Many studies discussed the recommended dosage for anti-cancer drugs, either in a single used drug or a combination of many drugs in an emulsion used for TACE [5]. The vast majority of interventional radiology centres are using doxorubicin adjusted to body weight doses in the range of 1 mg/kg [14]. Song et al. [6] performed a study on doxorubicin-loaded drug-eluting beads with a control group of conventional TACE; in the control group, the emulsion consisted of 3–15 mL of Lipiodol and 30–60 mg epirubicin or 40–60 mg doxorubicin, with the addition of cisplatin (40–70 mg) and/or 5-fluorouracil (130–250 mg). In a Japanese-Korean study on 99 patients, Ikeda et al. [28] described the conventional TACE procedure with the use of a maximum of 100 mg/patient for epirubicin (45 mg median dose), 70 mg/patient for doxorubicin (40 mg median dose), and 20 mL/patient for Lipiodol (5 mL median dose).

In our study, amongst the 28 included patients, 68 TACE procedures were performed. Based on the dose of doxorubicin, patients were randomised into group A (50 mg doxorubicin) or group B (100 mg doxorubicin). This is the first study that investigates the tolerability of different doses of doxorubicin in patients undergoing conventional TACE procedures as a treatment for primary HCC. However, the main limitation of our study was the small sample size of the included patients which limits the generalisation of our findings. Generally, patients with a large tumour size tend to suffer more from PES [29]. However, although in this study we did not find significant differences between the groups regarding the total size of the masses, we did not find a significant correlation between tumour size and severity of PES symptoms.

Several periprocedural methods have also been described to decrease post-procedural pain with narcotic administration, such as periprocedural intraarterial lidocaine and celiac plexus neurolysis, but none has proven to prevent the remaining symptoms of PES [12, 30]. Hepatic damage after TACE procedures may adversely affect the liver function and worsen the prognosis [22].

The main endpoint to study the benefit of techniques and methods for the treatment of HCC is OS and other methods of assessment including local response, time to progression of disease, and disease-free survival [31]. Another important endpoint for TACE, *i.e.*, the cumulative absence of fever, nausea, vomiting, and anorexia, can be considered as an important endpoint for TACE procedures [29].

Doxorubicin injection is generally painful; however, the post-injection pain severity varies according to the different pain threshold amongst patients. Usually, the direct ischemic effect of injection of Lipiodol/doxorubicin mixture to liver capsule induces pain during injection; pain during injection could be influenced by the release of some free particles of chemotherapy that did not combine with Lipiodol. Fever can be explained by the release of pyrogens from tissue breakdown into the blood, which affects the centres of temperature regulation in the brain. In this study, patients who received TACE with doxorubicin 50 mg demonstrated shorter duration and lower degree of pain compared with doxorubicin 100 mg TACE. All patients in this study had suffered from variable degrees of discomfort during the procedures, and peri-procedural administration of dexamethasone, pethidine, and ondansetron was fairly enough to control pain, nausea, and vomiting during the procedures. Intra-arterial injection of 1 mL of 2% lidocaine through the catheter with a maximum of 10 mL can help in achieving a rapid relief of intra-procedural pain.

Ascites, an indicator of worsened liver function, could have theoretically resulted from any direct insult to liver cells, such as the injection of cytotoxic agents during the TACE procedure. In this study, low-dose doxorubicin TACE did not show any increase in the incidence rates of ascites nor vomiting in comparison with the commonly used dose (100 mg) doxorubicin TACE. However, doxorubicin dose was directly proportional to the first week, VAS score, time to be free from PES, and vomiting.

Shin [7] concluded that the PES is a predictor of death and survival with a twofold increased risk of death in patients suffered from PES. PES is unavoidable; however, its control is thought to be associated with prolonged survival [21]. PES can interfere with the quality of life, and pre-medications can help control some symptoms. According to Hartrumpf et al. [21], the preprocedural medications for pain, vomiting, and fever to be continued after TACE can help decrease the PES after TACE. Many other studies showed that the response to TACE after the first and second sets was the same [24–26]. The addition of chemotherapy to Lipiodol in TACE is generally associated with more response and lower incidence rates of new angiogenesis, but embolisation alone can trigger tumour angiogenesis with failure of response [6, 27]; therefore, it is better to use chemotherapy in a dose that is sufficient to induce cytotoxicity with lower symptoms of PES. Many studies recommended that the doses range between 10 and 150 mg of doxorubicin per session [24, 28–31].

In a meta-analysis [32] including six randomised controlled trials comparing TACE and bland embolisation for treatment of HCC (totalling 676 patients, 342 treated with TACE and 334 with bland embolisation with no chemotherapeutic drug injected), non-superiority of either technique was found. TACE could be more harmful due to drug toxicity with more severe symptoms of PES. In addition, many authors considered that ischemia is playing the main lethal role to tumour cells more than the effect of drugs [33].

The most important endpoint in oncology practice and in HCC is OS [1]. Regarding survival after TACE procedures, many factors have shown an association with better OS. The reduction in hepatic enzymes, absence of distant spread, degree of parenchymal hepatic disease, homogenous Lipiodol uptake, and smaller tumour sizes are all associated with better survival in patients treated with chemoembolisation [8]. In this study, the change of doxorubicin dose between the two groups did not affect OS. In a single-centre randomised trial conducted by Brown et al. [34], the authors found no significant differences regarding OA and PES between the two groups of patients subjected to embolisation with either doxorubicin-eluting microspheres loaded by 150 mg doxorubicin or with microspheres alone. This could be similar to our results in the term of OA; however, we should conclude that many other factors could affect the severity and duration of PES mainly the toxic effect of chemotherapeutic drugs.

Our study has limitations, the main one being the small sample size of the included patients, which limits the statistical power of the comparisons. In particular, patients with CR at 1, 3, and 6 months were higher in group B with 100 mg doxorubicin, although the difference resulted to be not significant. As a consequence, further larger studies are recommended to evaluate the relation between doses of chemotherapeutic drugs used for intra-arterial injection in TACE and complete response at the first follow-up imaging study and furtherly. In addition, patients with large tumour size tend to suffer more from PES. Thus, evaluating response considering Lipiodol uptake could be misleading for the possible underestimation due to Lipiodol-induced artefacts. However, in this study, no data regarding the correlation of the size of the malignant lesion(s) and outcome results could be documented.

In conclusion, in TACE procedures, 50 mg doxorubicin showed fewer PES symptoms than 100 mg doxorubicin. The doxorubicin dose was positively correlated with post-injection VAS score, fever days, pain duration, and vomiting while no significant difference was observed for vomiting and ascite occurrence. Further, larger randomised controlled trials are required to support these findings, especially regarding complete response at the first follow-up imaging study and furtherly.

## Data Availability

The datasets used and/or analysed during the current study are available from the corresponding author on reasonable request.

## Abbreviations

ALT: Alanine aminotransferase
AST: Aspartate aminotransferase
CR: Complete response
CT: Computed tomography
EASL: European Association for the Study of the Liver
HCC: Hepatocellular carcinoma
INR: International normalised ratio
IQR: Interquartile range
mRECIST: Modified Response Evaluation Criteria in Solid Tumors
OS: Overall survival
PD: Progressive disease
PES: Post-embolisation syndrome
PR: Partial response
SD: Stable disease
VAS: Visual analogue scale
